# GH deficiency and insensitivity in children and adults

**DOI:** 10.1007/s11154-021-09629-1

**Published:** 2021-02-10

**Authors:** Gudmundur Johannsson, John J. Kopchick

**Affiliations:** 1 Institute of Medicine, Sahlgrenska Academy, University of Gothenburg, Gothenburg, Sweden; 2 Edison Biotechnology Institute, Ohio University, Athens, OH USA

**Keywords:** Growth hormone, IGF-I, Growth, Growth hormone deficiency, Growth hormone receptor, IGF-I receptor, Pharmacogenomics

## Abstract

This thematic review includes short reviews on GH deficiency and insensitivity in children and adults from basic science to clinical significance.

We are proud to present this thematic review on growth hormone (GH) deficiency and insensitivity in children and adults. It contains short reviews made by eminent experts in the field that together comprises a comprehensive assessment of the field from basic science to clinical significance.

The activity of the GH-IGF-I axis is regulated by the amount and secretory pattern of the GH, the endocrine and paracrine/autocrine production of IGF-I and the interaction of GH and IGF-I at the tissue specific level. Additionally, GH and IGF-1 have both unique and overlapping actions related to growth and metabolism. Therefore, overall effects of the GH-IGF-axis on growth and metabolism is dependent on the amount of GH and IGF-I at the tissue level as well as the responsiveness/sensitivity of the GH and IGF-I receptors.

Through clinical observation and experience, clinicians have observed variable responses to GH treatment in children. For example, the same GH exposure can produce a highly variable magnitude of growth. Similarly in adults, GH replacement in patients with hypopituitarism and severe GH deficiency can produce a response that is highly variable. In adults the efficacy of GH replacement varies from changes in body composition and lipid profile to improvement in well-being and quality of life. Additionally, it is also unpredictable to which of these endpoints an individual subject seems to respond most.

The aim of this thematic issue was therefore to shed light on the clinical aspects related to GH deficiency and insensitivity in both children and adults. This issue also includes human models of extreme GH insensitivity induced by inactivating changes in the GH receptor as well as at the post receptor level. The careful phenotyping as well as the molecular discoveries in these patients have extended the findings on the physiology of the GH-IGF-I axis and some of the individual sensitivities attributed to either GH or IGF-I. These observations have also been useful in better understanding the consequences of long-standing GH deficiency in children and adults. In addition to human studies, animal models of GH deficiency and insensitivity have also improved our understanding on the impact of GH and/or IGF-1 on tissue specific metabolism and in the end its impact on health- and life-span. Related to this is the findings in adults with GH deficiency that showed reduced survival in the unreplaced state. The causal relationship with GH deficiency has been debated and is further reviewed and discussed in this thematic issue.

The clinical challenges in the diagnosis of GH deficiency and various degree of insensitivity are further discussed together with strategies to improve outcome among these patients. With a perfect mix between basic, translational and clinical topics related to GH deficiency and insensitivity, we hope that the readers of this issue will enjoy and appreciate the science behind the short reviews. We also hope that the reader will improve their understanding of the molecular mechanism(s) of GH/IGF-1 action, the clinical consequences and the treatment strategies to improve each patient’s outcome from childhood to adulthood.

